# Anterior Protrusion of Hydroxyapatite Blocks During Vertebroplasty for Thoracic Burst Fractures: A Case Report

**DOI:** 10.7759/cureus.58784

**Published:** 2024-04-22

**Authors:** Yuko Yagi, Chiaki Horii, Shimpei Koyama, Yuki Onishi, Naohiro Kawamura

**Affiliations:** 1 Department of Spine and Orthopedic Surgery, Japanese Red Cross Medical Center, Tokyo, JPN; 2 Department of Orthopedics, Tokyo Metropolitan Hiroo Hospital, Tokyo, JPN

**Keywords:** complication, percutaneous stabilization, burst fracture, vertebroplasty, hydroxyapatite block

## Abstract

Research on complications necessitating reoperation following vertebroplasty related to hydroxyapatite (HA) blocks is limited. We present the case of a 25-year-old woman who underwent posterior fixation and vertebroplasty using HA blocks for a T12 burst fracture. Postoperative computed tomography revealed anterior protrusion of some blocks, with consequent compression of the descending aorta. We removed the protruded blocks viaa transthoracic approach and observed no aortic injuries. Although HA blocks are considered safe for vertebroplasty, surgeons should be aware of the risk of anterior protrusion and potential aortic injury.

## Introduction

Surgical treatment for thoracolumbar burst fractures aims to stabilize the spine and enable the early mobilization of patients while preventing neurological deficits. Short-segment posterior fixation with percutaneous pedicle screws (PPSF) is widely accepted as a good treatment option for thoracolumbar burst fractures because it can achieve enough stabilization with minimal invasion [1]. Some surgeons perform vertebroplasty of the fractured vertebra in addition to PPSF, especially when the fractured vertebra is severely damaged [2-5]. Vertebroplasty has been reported to reduce implant failure and improve clinical outcomes in some studies [4,5], while others have reported no difference in outcomes between procedures performed with and without vertebroplasty [2,3]. It remains controversial whether vertebroplasty, in addition to PPSF, should be conducted when treating thoracolumbar burst fractures.

Some studies have reported complications related to the material used for vertebroplasty. Liquid materials, such as polymethylmethacrylate (PMMA) and calcium phosphate paste, have been commonly used in vertebroplasty; however, they may leak from the vertebra and cause pulmonary embolism, paraplegia, and other complications [6]. Furthermore, PMMA is reportedly associated with subsequent vertebral fractures, especially in adjacent levels. The discrepancy in strength between the treated and other vertebrae can lead to subsequent fractures [7]. Therefore, materials with stiffness closely resembling that of the bone are preferred for vertebral augmentation procedures.

Matsuzaki first reported performing vertebroplasty using solid hydroxyapatite (HA) blocks to overcome these complications [8]. As HA blocks are not liquid, they do not easily leak from bones or migrate into vessels. Furthermore, HA grafts have osteoconductive properties; thus, they unite with the surrounding bone over weeks to months [9]. Therefore, vertebroplasty with HA blocks is considered safer compared to procedures with other materials, and only a few studies have reported complications associated with HA blocks [10-12]. Here, we present a rare case wherein HA blocks protruded anteriorly and caused significant aortic compression.

This article was previously presented at the 30th Annual Meeting of the Japan Society for the Study of Surgical Technique for Spine and Spinal Nerves on September 15, 2023.

## Case presentation

**Figure 1:**
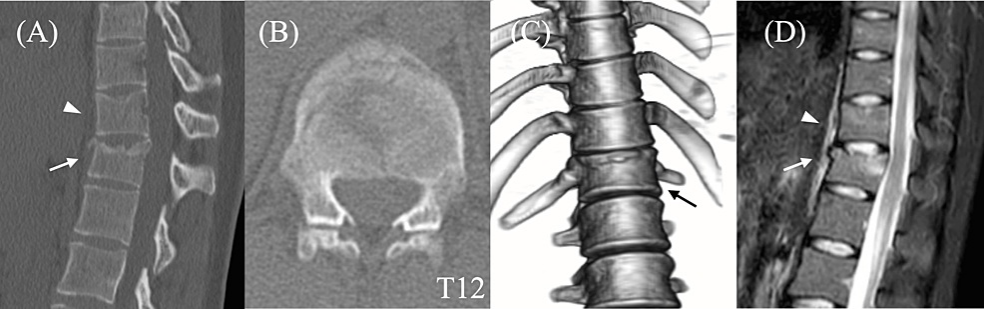
Preoperative images of the thoracolumbar spine. (A) Sagittal computed tomography; (B) axial computed tomography; (C) three-dimensional reconstruction image; and (D) sagittal short TI inversion recovery sequence in magnetic resonance imaging. T11 compression fracture (arrowhead) and T12 burst fracture (arrow).

A 25-year-old woman jumped from the third floor of a building and was brought to our hospital in an ambulance. She complained of back pain but did not show any neurological deficits. The patient was diagnosed with T11 AO-type A1 compression and T12 AO-type A3 burst fractures (Figures 1A-1C). The AO Spine classification is a system for classifying thoracolumbar fractures into nine types: A0 to A4, B1 to B3, and C types. Type A indicates compression injuries; type A1 indicates wedge-compression fractures, whereas type A3 indicates incomplete burst fractures with superior or inferior end plate alone being involved. Type A3 fractures are usually treated with short-segment posterior fixation. Magnetic resonance imaging indicated the absence of spinal canal compromise, posterior ligament complex injury, or clear evidence of anterior longitudinal ligamentous rupture (Figure 1D). Consequently, PPSF with vertebroplasty at T12 was scheduled to facilitate early mobilization. 

**Figure 2:**
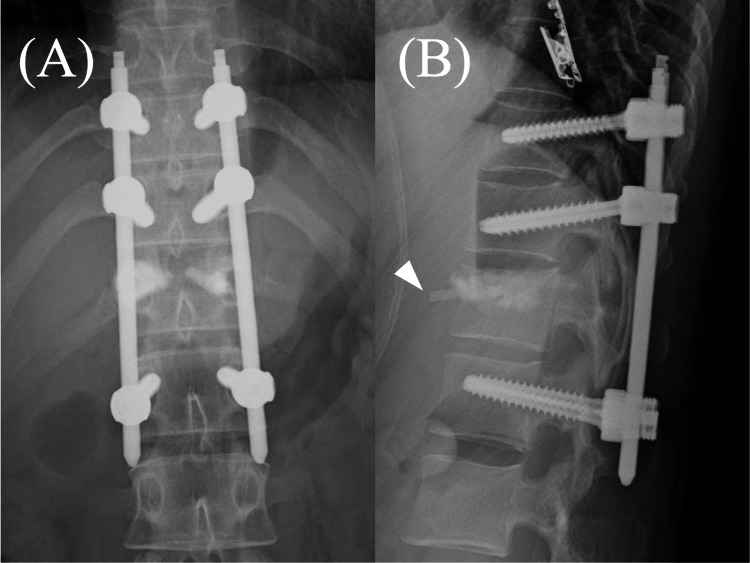
Postoperative radiographs: (A) anterior-posterior view and (B) lateral view. Anterior protrusion of hydroxyapatite blocks (arrowhead) is observed in the lateral view.

Percutaneous pedicle screws (PPSs) were inserted at T10, T11, and L1. Indirect reduction was performed via ligamentotaxis using these screws; direct transpedicular reduction was performed for the superior end plate at T12 using dedicated elevators, thereby creating a void in the vertebra to be filled with HA blocks. After positioning an inserter into the vertebra using fluoroscopic guidance, HA blocks were inserted into the void. The insertion process initially commenced on the right side, with subsequent insertion on the left side. However, shortly after initiating the filling process on the left side under continuous fluoroscopic guidance, it became apparent that the blocks had protruded anteriorly. Therefore, insertion was halted, rods were connected, and surgery was completed (Figure 2). In total, we inserted 42 blocks on the right side and nine blocks on the left. Vital signs remained stable during the procedure.

**Figure 3:**
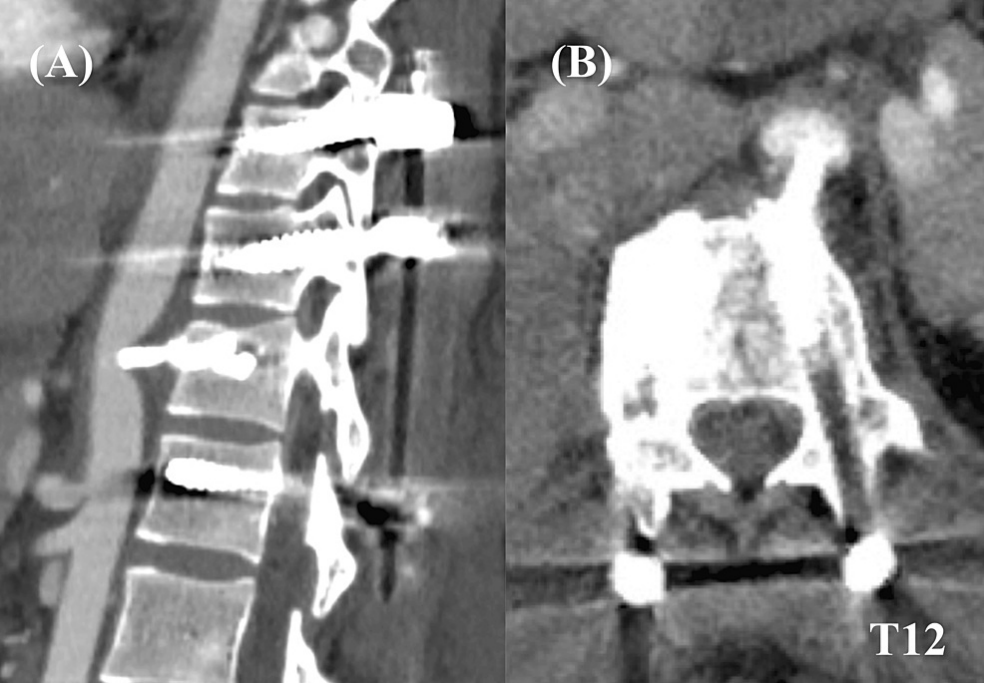
Postoperative contrast-enhanced computed tomography: (A) sagittal view and (B) axial view. HA blocks are observed compressing the descending aorta. HA, hydroxyapatite

**Figure 4:**
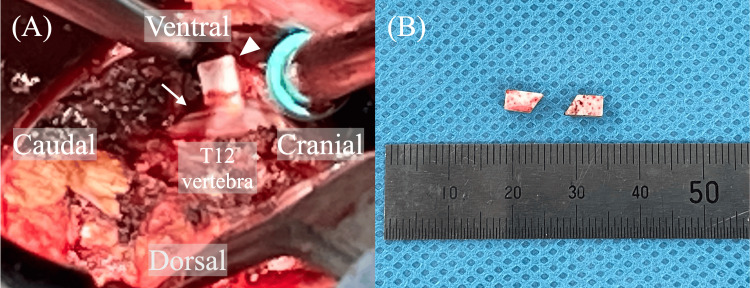
Interoperative image. (A) A hydroxyapatite block (arrowhead) is observed stuck to the anterior longitudinal ligament (arrow). (B) The hydroxyapatite blocks were removed during the reoperation.

Postoperative contrast-enhanced computed tomography revealed that two blocks had compressed the descending aorta without periaortic contrast-medium extravasation (Figure 3). These were scheduled for removal on postoperative day 3. In preparation for potential aortic injury, we consulted a cardiovascular team and radiologists; they inserted a sheath into the femoral artery immediately before the removal surgery to prepare for stenting or intra-aortic balloon occlusion. The T12 vertebra was explored using the usual transthoracic approach. Following ligation of the left segmental artery on the T12 vertebra, the anterior soft tissue, including the aorta, was cautiously retracted anteriorly from the T11-12 disc level to the T12-L1 disc level, facilitating visualization of the anterior space of the T12 vertebra. Upon examination, the first block was observed anterior to T12 and adhered to the anterior longitudinal ligament, whereas the second block was situated beneath the first (Figure 4). No aortic bleeding was observed. The patient was discharged on postoperative day 11.

## Discussion

We presented a case of vertebroplasty performed using HA blocks that protruded anteriorly and compressed the aorta. The protruded blocks were removed via the transthoracic approach, and no aortic injury was observed.

Compared with other conventional materials, such as PMMA and calcium phosphate paste, HA blocks are considered safe for vertebroplasty, although block protrusion is not uncommon (9.4%-35.5%; anterior protrusion 4.3%) [11-13]. Extravertebral protrusion can lead to spinal cord compression [11] and pulmonary embolism [10]. However, to the best of our knowledge, no cases of HA block-associated aortic compression or injury have been reported to date. Aortic injury is a life-threatening complication, the results of which can be devastating. All surgeons should be aware of the risk of aortic injury associated with vertebroplasty and be familiar with the means to prevent it.

In the present case, the anterior protrusion of HA blocks occurred shortly after the initial step of block insertion, despite careful and continuous fluoroscopic guidance. Thus, it is unlikely that block protrusion resulted from excessive packing. We speculated three possible reasons for the protrusion. First, an anterior wall rupture might have expanded during reduction. Preoperative computed tomography scanning showed the depressed superior end plate of the T12 vertebra, which did not constitute a craniocaudal cleft in the anterior wall. However, as we performed indirect and direct reduction maneuvers before vertebroplasty, a cleft might have been created in the anterior wall. Furthermore, our examination of the anterior wall using elevators during transpedicular reduction failed to reveal the presence of an anterior cleft. This may be attributed to the difference in size between the elevators and HA blocks; the heads of the elevators were larger than those of the HA blocks; thus, some clefts could have been missed. Second, massive void formation in the fractured vertebra was difficult because the patient was young with good bone quality, and the degree of fracture comminution was relatively minor. Posterior stabilization alone without vertebroplasty might have been sufficient in this case. Third, the HA block inserter may have been positioned too anteriorly for this particular case (wherein massive void formation was difficult).

In general, posterior positioning of the inserter should be avoided to prevent intra-spinal-canal leakage. Moreover, anterior positioning should also be avoided in vertebrae with minimum void. Surgeons should recognize the risk of anterior protrusion during vertebroplasty using any material as it may cause aortic injury. We suggest the careful insertion of HA blocks, particularly at the outset of the procedure, with the inserter positioned appropriately to avoid excessive anterior placement. Once some blocks are inserted and an anterior barrier is created, the risk of anterior protrusion may be reduced.

## Conclusions

In conclusion, we presented the case of a patient who underwent reoperation after vertebroplasty due to anterior protrusion of HA blocks. When HA blocks protrude anteriorly, they may compress the aorta and lead to aortic injury. Careful preparation would be needed for reoperation to remove HA blocks protruded anteriorly. Surgeons should consider the position of the block inserter carefully to avoid such a protrusion.
